# Maternal Responses and Adaptive Changes to Environmental Stress via Chronic Nanomaterial Exposure: Differences in Inter and Transgenerational Interclonal Broods of *Daphnia magna*

**DOI:** 10.3390/ijms22010015

**Published:** 2020-12-22

**Authors:** Laura-Jayne. A. Ellis, Stephen Kissane, Iseult Lynch

**Affiliations:** 1 School of Geography, Earth and Environmental Sciences, University of Birmingham, Birmingham B15 2TT, UK; L.A.Ellis@bham.ac.uk; 2 School of Biosciences, University of Birmingham, Birmingham B15 2TT, UK; S.Kissane@bham.ac.uk

**Keywords:** nanomaterials, daphnia magna, epigenetics, environmental toxicology

## Abstract

There is increasing recognition that environmental nano-biological interactions in model species, and the resulting effects on progeny, are of paramount importance for nanomaterial (NM) risk assessment. In this work, Daphnia magna F0 mothers were exposed to a range of silver and titanium dioxide NMs. The key biological life history traits (survival, growth and reproduction) of the F1 intergenerations, at the first (F1B1), third (F1B3) and fifth (F1B5) broods, were investigated. Furthermore, the F1 germlines of each of the three broods were investigated over 3 more generations (up to 25 days each) in continuous or removed-from NM exposure, to identify how the length of maternal exposure affects the resulting clonal broods. Our results show how daphnids respond to NM-induced stress, and how the maternal effects show trade-offs between growth, reproduction and survivorship. The F1B1 (and following germline) had the shortest F0 maternal exposure times to the NMs, and thus were the most sensitive showing reduced size and reproductive output. The F1B3 generation had a sub-chronic maternal exposure, whereas the F1B5 generation suffered chronic maternal exposure where (in most cases) the most compensatory adaptive effects were displayed in response to the prolonged NM exposure, including enhanced neonate output and reduced gene expression. Transgenerational responses of multiple germlines showed a direct link with maternal exposure time to ‘sub-lethal’ effect concentrations of NMs (identified from standard OECDs acute toxicity tests which chronically presented as lethal) including increased survival and production of males in the F1B3 and G1B5 germlines. This information may help to fine-tune environmental risk assessments of NMs and prediction of their impacts on environmental ecology.

## 1. Introduction

Nanotechnology, which is the ability to manipulate materials at the nanoscale in order to exploit the advanced functionality and novel properties of engineered nanomaterials (NMs), has revolutionised the materials science leading to an enormous range of industrial and consumer applications. The diversity of compositions, sizes, shapes and surface functionalisation available at the nanoscale, coupled with their interactions with biological molecules and cellular machinery have made NMs a useful tool in diagnostics (as contrast agents) and for drug delivery wherein they can exploit endogenous transport mechanisms to deliver therapeutics to target areas [1,2,3]. Properties such as large surface areas, surface activity and specific affinity of organic based NMs and zero-valent transition-metal NMs have seen a beneficial impact on environmental challenges, such as the detection and removal of inorganic heavy metals and organic and other inorganic contaminants from polluted water and soils [4,5]. NMs such as silica and silver (Ag) have also been developed for the agricultural and forestry industries as part of nanopesticide formulations [6]. Other properties including optical, thermal and photocatalytic are also enhanced at the nanoscale, such that photocatalytic materials like titanium dioxide (TiO_2_) have been utilised in the electronic and energy producing industries [7,8].

In addition to their aforementioned industrial applications, Ag and TiO_2_ NMs are also commonly used in consumer applications, particularly within the healthcare industries [9] and are added to cosmetic products [10]. The incorporation of NMs into such products exploits the antibacterial and anti-microbial properties of Ag NMs [11] and the photoelectronic properties of TiO_2_ NMs which have the ability to block the UV radiation from sunlight and act as sun protection factors (SPFs) [12,13]. Although these technological advances are important, the resulting environmental repercussions, when NMs are discharged into the environment (and thus become pollutants) and the resulting organism interactions and broader impacts on the environment need to be addressed in parallel to development of applications.

Aquatic organisms have the ability to cope with an array of variable environmental conditions, such as temperature, predator stress and food levels, each of which influences an organism’s life history traits including survivorship, growth and reproduction [14,15]. Moreover, information about the environmental stress can be transferred to the progeny as a maternal phenotypic response [15]. Therefore, chronic environmental stress over multiple generations may lead to a genetically diverse natural population resulting in the natural selection of more tolerant individuals, i.e., individuals that can maintain fitness under the specific environmental stress [16].

*Daphnia* are freshwater crustaceans and are among the relatively few organisms that reproduce parthenogenetically [17], making daphnids an excellent candidate for studying environmental influences on epigenetic developmental programs. Most importantly, in the context of epigenetics, while the offspring are clonally (genetically) identical, they may not be epigenetically identical. Under stressful conditions induced by environmental cues, clonal diploid eggs can develop as males rather than females [17,18]. Monitoring deviations from normal development under environmental stress provides insights into how environmental changes influence daphnids epigenetic repertoire [17]. 

Epigenetic changes occur as a result of modifications of the histone proteins of chromatin and DNA methylation, resulting in altered gene expression [19,20]. DNA methylation modulates the activation and inactivation of genes, which changes the phenotype, without altering the genome [21]. Epigenetic markers are influenced by an array of environmental factors, including stresses arising from chemical exposure, malnutrition and developmental cues [18,21,22]. There is a significant body of evidence to support phenotypic alterations as a means to enhance an organism’s ability to adapt and respond to environmental stress and thrive via transgenerational inheritance, where the progeny displays altered phenotypic characteristics as a direct result of maternal stress [22,23,24]. Generational phenotypes result from direct toxicity and the physiological effects of exposure [22,25,26]. Toxicant exposure to the parent F0 generation, therefore, may result in a direct effect on the F1 generation and their subsequent germlines [27]. However, if the germline is not affected, no phenotypical effects will be observed in the subsequent generations. Therefore, maternal stress in *Daphnia* can be used to identify transgenerational epigenetic inheritance of the phenotype altered as a response to changing environments [22]. Furthermore, the number of genes showing sensitivity or adaptation to environmental or toxicant stress should increase with increasingly stressful conditions or extended exposure times, and be presented as phenotypical changes within the offspring [28].

The responses of *Daphnia magna* to stress have been well documented for both chronic and acute exposures, focusing on longevity and reproduction [29,30,31]. However, the majority of studies fail to consider the effects on developmental time and transgenerational inheritance of the maternal stress between the interclonal broods born of the same exposed mother (the intergenerations), nor do they follow the germlines of each of these broods (the trasngenerations). The present study identifies the environmental influence across multiple generations from both continuous and F0 generation only exposure of *Daphnia magna* to TiO_2_ and Ag NMs. The adaptation/tolerance to the chronic maternal stress via phenotypical variations over time were assessed by monitoring the differences between the intergenerations of the first, third and fifth F1 clonal broods (born of the same NM-exposed mother). Offspring from each of the three broods were either kept in continuous exposure to the NMs or removed (the F1 generation is removed within 24 h of birth and further cultured in fresh NM-free medium to assess the potential for recovery from the material exposure), and monitored for fitness within each of their transgenerational germlines. 

Since the first F1 broods (F1B1) have the shortest maternal exposure times to the NMs, we expect the most sensitivity in these broods as the mother (F0) gets used to the NM containing environment. The third broods (F1B3) would have a subchronic exposure (a median between the first and fifth broods), whereas the fifth broods (F1B5) would have the longest (chronic) maternal exposure. We therefore expect the fifth F1 broods and their transgenerational germlines to show more tolerance to the NMs in both the continuously exposed and recovery daphnids. Since exposure duration is important in shaping biological responses [32], we exposed *Daphnia magna* neonates (that form the F0 parents), at ≤ 24 h old for between 24–36 days (until the fifth F1 broods were born) and monitored their survival, growth, reproduction and gene expression. The germlines of F1 broods 1, 3 and 5 were subsequently tracked over three additional generations using a paired continuous exposure versus recovery approach to assess epigenetic effects. 

## 2. Results and Discussion

### 2.1. Nanomaterial Characterization 

The NMs were pre-characterised before *Daphnia* exposures [24] and the results are presented in Table 1 and Figure S2 (Supporting information).

### 2.2. Longevity and Growth Effects

**Figure 1:**
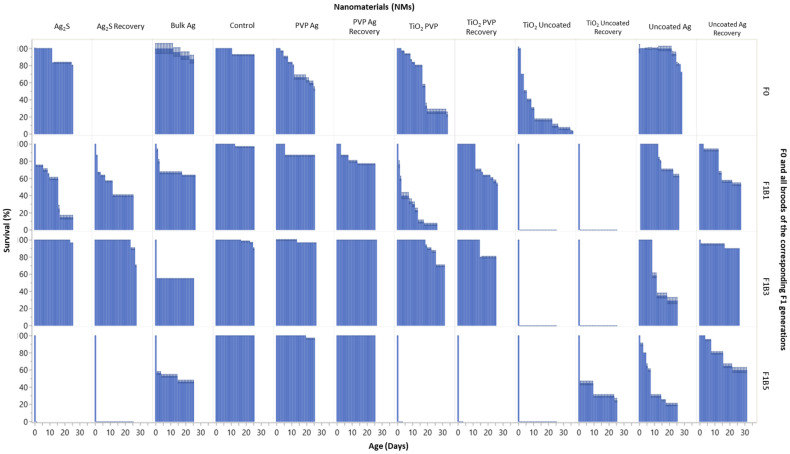
Survival (%) versus age (days) for all F0 generations and broods F1B1, F1B3 and F1B5 of the subsequent F1 generations. Data are presented for daphnids continuously exposed to each of the Ag and TiO_2_ NMs, and those in the recovery sets after removal from exposure (*n* = 5). The *Y*-axis indicates the average daphnid survival (%) as a function of daphnid age (days) on the *X*-axis. F0 = Parent exposure to the particular NM are noted at the top of the plots.

**Figure 2:**
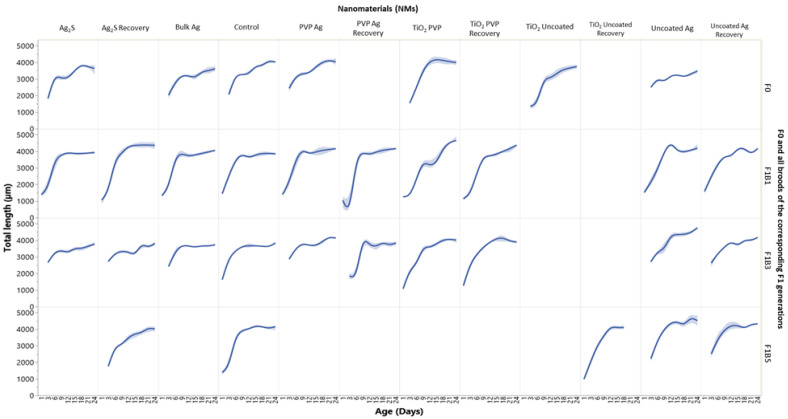
Size (measured as tail length) versus age for all F0 generations and broods F1B1, F1B3 and F1B5 of the subsequent F1 generations. Data are presented for daphnids continuously exposed to each of the Ag and TiO_2_ NMs, and those in the recovery sets after removal from exposure. The *y*-axis indicates the average daphnid length (µm) (measuring from the apex of the helmet to the base of the tail) as a function of the daphnid age (days) on the *X*-axis. F0 = Parent exposure to the particular NM is noted at the top of the plots. The graph splits horizontally by each of the generations and vertically by the NM exposure condition. The shaded areas around the lines are the 95% confidence bands. N = 5.

To understand the effects of maternal exposure to NMs on the subsequent progeny, we must first assess the F0 parent generation response to the NMs. Over the study duration (between days 24–36), the F0 parent generations (Figure 1) had a total survival of 100% (uncoated Ag NMs), 53% (PVP Ag NMs), 80% (Ag_2_S NMs), 88% (Bulk Ag), 10% (TiO_2_ uncoated) and 27% (TiO_2_ PVP). Survival was coupled with reduced overall daphnid body lengths, which were on average 12% (uncoated Ag), 8% (Ag_2_S), 5% (Bulk Ag), 48% (uncoated TiO_2_) and 23% (PVP TiO_2_) smaller than the controls over the duration of the study (Figure 2). There were no significant body length differences for the F0 generation exposed to the PVP Ag NMs. The F1B1 broods, which were produced on day 11 of the maternal exposure (PVP Ag NMs) had the shortest maternal exposure times to the NMs, whereas the F1B3 broods, produced on day 18, had sub-chronic maternal NM exposures (a median between the first and fifth broods), while the F1B5 generations born on day 24 had chronic maternal NM exposure. Thus, a comparison of the intergenerational responses and subsequent transgenerational effects within the three brood germlines may provide important insights into the organisms’ responses to NMs exposure over chronic exposure times.

The effects of the maternal exposure duration were evident in the differential sensitivity and survival exhibited across the three intergenerational F1 broods. For example, daphnids continuously exposed (_exp_) to the uncoated Ag NMs in the F1B1_exp_ generation had 63% survival, compared with only 29% (F1B3_exp_) and 20% (F1B5_exp_) survival at day 25 in the later broods. The F1B1 generation were born when the F0 parent was 13 days old, compared with 22 days old when the third broods (F1B3) were produced, and 25 days old when the fifth broods (F1B5) were born. The prolonged maternal exposure resulted in more severe effects on the later broods of the F1 intergenerations. Observations of the F2 generations in the F1B1_exp_ germline (Figure S3) exposed to the uncoated Ag NMs further show that the daphnids in continuous exposure did not survive. However, the F2 and F3 transgenerations in the F1B3_exp_ and F1B5_exp_ germlines exposed to the same NMs (Figures S4 and S5) thrived, showing increased adaptive measures based on prolonged maternal exposure. By contrast, the recovery (_rec_) intergenerations for the uncoated Ag NM exposure set (whereby the F1 daphnids were removed to NM free medium) had 53% (F1B1_rec_), 90% (F1B3_rec_) and 60% (F1B5_rec_) survival by day 25. With the exception of the F1B1 generation, the F1 recovery generation (F1_rec_) had significantly increased survival compared with the three broods of the F1_exp_ generation left in continuous exposure to uncoated Ag NMs. The daphnid survival also increased in the following F2_rec_ intergenerations of the F1B1_rec_, F1B3_rec_ and F1B5_rec_ germlines of the recovery transgenerations. Figures S3–S5 (Supporting information) show the survival (%) of subsequent generations following the F1B1, F1B3 and F1B5 germlines taken from the third broods of each generation for both exposure and recovery daphnids. 

In parallel with their effects on the F0 parent generation, less severe effects on the survival of the daphnids exposed to the PVP Ag NMs were observed between the three intergenerational F1 broods (as compared to uncoated Ag NMs). Daphnids in the F1B1_exp_ generation had 87% survival, and those in the F1B3_exp_ and F1B5_exp_ generations had 97% and 94% survival at day 25, respectively. The recovery intergenerations also demonstrated regeneration, showing 77% (F1B1_rec_) and 100% (F1B3_rec_ and F1B5_rec_) survival. Transgenerational monitoring was not conducted for the F1B1 germline, however, the F1B3_exp_ germline for the daphnids continuously exposed to PVP Ag NMs did show sensitivity in both the F2 and F3 transgenerations, with no surviving daphnids in the F3_exp_ generation (Figure S4). However, daphnids were less sensitive in the exposed F1B5_exp_ germline with 67% and 93% survival in the F2_exp_ and F3_exp_ generations (Figure S5). These data suggest that as the F0 maternal exposure duration increased adaptive mechanisms were activated, which were passed onto the F1B5 transgenerational germline. Despite the low NM concentration used, daphnids were highly sensitive in the F1 intergenerations when exposed to TiO_2_ uncoated NMs, with no surviving F1B1 and F1B3 generations for either the continuously exposed or recovery exposures. However, continuous exposure of the F0 daphnids appears to induce adaptive measures since the F1B5_rec_ generation did have 25% surviving offspring at day 25. 

Developmental abnormalities in terms of growth were also observed between the first, third and fifth broods of the F1 intergenerations. On average for the F1B1_exp_ generation, most exposures resulted in a reduction in the average daphnid sizes (5% uncoated Ag; 3% Ag_2_S; 4% Bulk Ag; 22% TiO_2_ PVP; and 18% TiO_2_ uncoated) when compared to the controls (Figure 2). By contrast, daphnids exposed to the PVP Ag NMs were on average 3% larger than the controls. The most severe effects on growth in the F1B1_exp_ generation were observed in the daphnids exposed to the TiO_2_ PVP NMs which were 22% smaller when compared to the unexposed controls. These results were also mirrored for the F1B3_exp_ and F1B5_exp_ generations. The F1B3_rec_ and F1B5_rec_ growth trends were more comparable with the control daphnids, for the exposed and recovery sets, with the most severely affected daphnids being those exposed to the uncoated Ag and TiO_2_ NMs. The results here show negative effects on the daphnid growth and development between the differently coated Ag and TiO_2_ NM exposures. The complete transgenerational observations (F2–3) for the growth trends of the F1B3 germline for all NMs (exposed/recovery) are shown in Figure S6.

Differences in the daphnid survival were observed between the intergeneration F1 broods with increasing maternal exposure duration. Additionally, daphnid sensitivity was mediated by the core speciation of the NMs and surface coating. In the presence of both Ag and TiO_2_ NMs the *Daphnia* suffered from stunted growth in comparison to the controls. Ag NMs dissolve in high ionic strength media such as the HH combo culturing medium [33,34]. In addition both metals (Ag and TiO_2_) can transform after accumulation in the gut and digestive tract, since the gut is acidic (~pH 4) which may impact the *Daphnia’s* energy budget due to impaired nutritional assimilation [35], which also explains the increased mortality and decreased body size in the present study. The results provide initial evidence to support the hypothesis that when daphnids are continuously exposed to toxicants (NMs) they switch on adaptive mechanisms, as exhibited by the variations of sensitivity between the intergeneration F1 broods and their following transgenerational germlines. 

### 2.3. Reproduction

**Figure 3:**
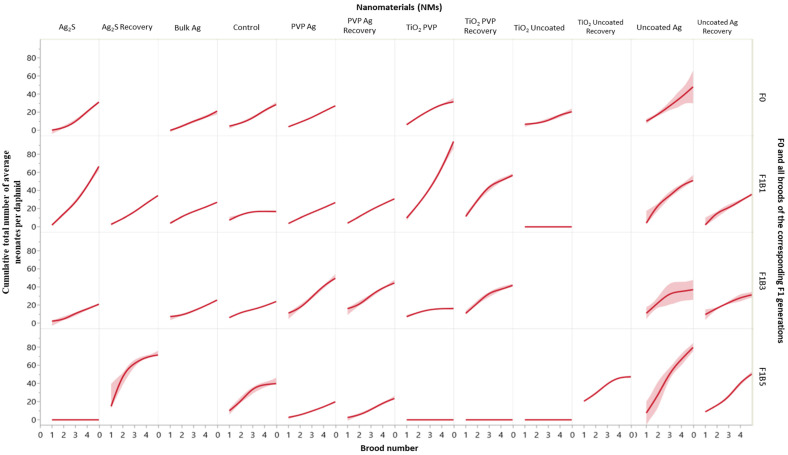
The average cumulative total neonates per daphnid for each brood (1–5), for all F0 generation and broods F1B1, F1B3 and F1B5 of the subsequent F1 generations. Data are presented for daphnids continuously exposed to each of the Ag and TiO_2_ NMs, and those in the recovery sets after removal from exposure. The *Y*-axis indicates the average cumulative total neonates per daphnid with increasing brood number on the *X*-axis. F0 = Parent exposure to the particular NM is noted at the top of the plots. The graph is split horizontally by each of the generations and vertically by the NM exposure condition. The shaded areas around the lines are the 95% confidence bands.

In stable environments, *Daphnia* typically start to reproduce between 5–10 days old, generating a clutch of parthenogenetic neonates approximately every 3–4 days thereafter [36]. Figure 3 shows the average cumulative total neonates per daphnid for each of the F0, F1B1, F1B3 and F1B5 intergenerational broods, and Figures S7–S9 and Tables S8–S10 (Supporting information) show the reproductive responses for each of the transgenerational germlines for the three monitored F1 broods (continuously exposed and recovery). 

The maternal F0 generations exposed to Bulk Ag, PVP-coated Ag NMs and Ag_2_S NMs released their first broods (which became the F1B1 generation) on day 11 in alignment with the brood timings of the control daphnids. Brood timings remained aligned with the controls for each of the subsequent broods for each of the germlines for these exposures (Figures S7–S9; Tables S8–S10). The average number of offspring per control daphnid (F0) was around 6–10 neonates per brood (Tables S8–S10; Figure 3), which was comparable with other studies using the same strain of *Daphnia* [37]. It is well known that the condition of the mothers has a significant influence on the phenotypic response of their neonates and their ability to adapt to environmental conditions [24,26,38]. Maternal exposure to uncoated Ag NMs, TiO_2_ uncoated and TiO_2_ PVP NMs had the most negative effects on the F0 reproductive cycles, with the first broods (forming F1B1) delayed until days 12 (PVP TiO_2_), 13 (uncoated Ag) and 17 (uncoated TiO_2_). The F0 generation exposed to uncoated Ag NMs, and TiO_2_ PVP NMs had an increased average of 20 neonates per brood (Figure 3). Similarly, studies by Agatz [29] identified a shift in the distribution of individual daphnid energy reserves, with reduced growth and increased reproduction in *Daphnia magna* after chronic exposure to environmental xenobiotics. 

The F1B1 generation (Table S8 and Figure S7) of daphnids exposed to the uncoated Ag NMs were born after the mothers (F0) were exposed for 13 days. The F1B1_exp_ generation exhibited subtle differences between their brood timings (forming F2) compared to the control populations (± 1 day). However, the average cumulative number or neonates per daphnid over the five F1 broods (producing the F2 generation) was 50, compared to only 28 in the controls (comparison with F0 control as data missing for the last two broods in the control F1B1) and 36 for the F1B1_rec_ generation (removed from uncoated Ag NM exposure at birth) (Figure 3). The F1B3 generation were born (4 days later than controls) after the mother (F0) had been exposed for 22 days to uncoated Ag NMs and were also 46% male. Investigating the F1B3 transgenerational germline identified that the third broods of the F2_rec_ set were also 26% male (forming the F3_rec_ generation), with approximately 18% of the F3_rec_ generation and 3% of the F3_exp_ generations (forming F4) being male. A similar pattern emerges for the F1B5 generation, which were also born after chronic maternal exposure (28 days), of which approximately 48% were male. However, no males were identified in the subsequent generations (exposed or recovery) following the F1B5 transgenerational germline. Male production is directly linked with maternal environmental stress [39], and is evident in the F1B3 and F1B5 generations after 22 and 28 days of maternal exposure to the uncoated Ag NMs. It is well documented that poor conditions and elevated stress levels are key precursors for female *Daphnia* to produce the males required for sexual reproduction [36]. An increase in male production decreases the output of parthenogenic females, and reduces population abundance [40], which was evidenced in the present study. The F1B3 germline (for both exposed and recovery generations) had noticeably more delays between offspring timing and a lower average number of neonates per daphnid compared to the F1B3 controls (Table S9). For the daphnids continuously exposed in this transgenerational germline, the most severe effect was in the F3_rec_ generation, which failed to produce fifth broods and did not become gravid thereafter. 

By contrast, daphnids exposed to the uncoated TiO_2_ NMs did not produce successive transgenerations for the F1B1 or F1B3 (exposed and recovery) germlines, while the F1B5_exp_ generation died within 24 h of birth. These adverse effects were due to the significant decrease in the F0 adult populations (3% survival overall by the time the F0 parents had brood 5). However, the F1B5_rec_ generation was gravid and successfully produced F2-F4 transgenerations (Figure S9; Table S10). The reproductive delays can also be associated with stunted growth in the exposed daphnids [30], as evidenced by the growth curves of daphnids in the uncoated Ag, TiO_2_ PVP and uncoated TIO_2_ NM exposures. The impact of the duration of maternal exposure to the TiO_2_ PVP NMs on the survival of the offspring was also exhibited between the F1 intergenerations where the most sensitivity was observed in the F1B5 generation, which were born after 33 days of maternal exposure (Table S8). Irrespective of continuous exposure to TiO_2_ PVP NMs or removal and recovery, all F1B5 daphnids died prior to achieving reproduction.

Differences in surface coating will determine the Ag NM stability and solubility in the medium [41,42]. Uncoated Ag NMs are more surface reactive and will dissolve more readily than those with strongly bound coatings like PVP as shown in previous investigations, suggesting strong effects from Ag^+^ [24,43,44]. Both surface coating and core material contributed to the observed toxicity of these NMs with lasting effects on the offspring in the subsequent germlines of the three different measured F1 broods. These NM exposures (uncoated Ag, uncoated TiO_2_ and PVP TiO_2_ NMs) were severely toxic and had the most negative effects on daphnid growth, longevity and reproduction throughout the exposed generations and germ lines, with some evidence of adaptation in the F1B5 germlines (both exposed and recovery after chronic maternal exposure). 

We further hypothesise that the internalization and accumulation of Ag and TiO_2_ NMs leads to prolonged oxidative stress in the *Daphnia* and reduced feeding, resulting in reduced fitness, survival and the inability to become gravid which was seen in later transgenerations of the same germlines [15,45,46], all of which were observed in the current study.

### 2.4. Gene Expression

The genes analysed in this study were chosen based on their specificity to NM exposure as reported in previous studies using *Daphnia magna* [47] and for their species diversity as biomarkers for cell viability/maintenance, oxidative stress, DNA repair and energy metabolism [47,48,49]. Measuring the gene expression of specific heterogeneous genes may indicate similarities between organism responses to the NM exposure and how information about maternal stress (and adaptation to environmental stress) can be inherited by the progeny [25,50,51]. 

**Figure 4:**
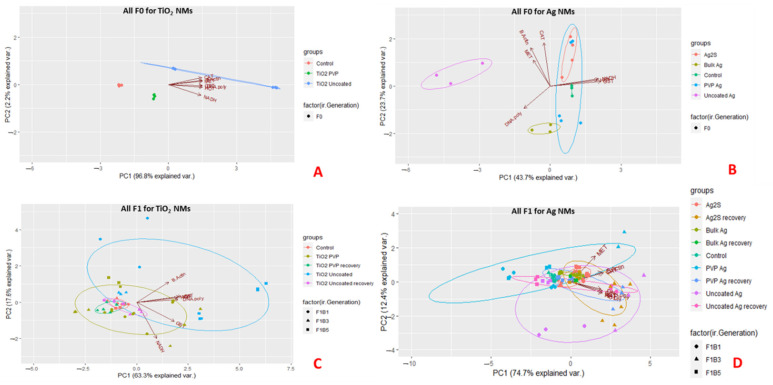
2D Principal Components Analysis (PCA) variable biplots showing the variation of the exposed and recovery broods responses to the NMs, plotted using their projections onto the first two principal components, combined with the gene expression plotted using their weights for the components. The red arrows indicate the direction of maximum loading of each gene to the overall distribution. The circles (Ag NM treatments only) show the F0 parents, the triangles show the first F1 brood (F1B1), squares show the third F1 brood (F1B3) and the crosses show the fifth F1 broods (F1B5). The colours represent each of the NMs. The PCAs are split into: (**A**) F0 generations exposed to the TiO_2_ NMs; (**B**) All exposed and recovery broods of the F1 generations for the TiO_2_ NMs; (**C**) F0 generations exposed to the Ag NMs; and (**D**) All exposed and recovery broods of the F1 generations for the Ag NMs. PCAs for each of the F1 germline generations (F2-4) are provided in the supporting information. Key = Glutathione S-transferase (GST), dehydrogenase (NADH), β-Actin (B-Actin), catalase (CAT), metallothionein (MET), DNA Polymerase (DNA-poly) and heme-oxygenase-1 (HO1). All genes are normalised to 18S ribosomal RNA (18S).

To assess the difference of the gene expression profiles between the interclonal broods, and their subsequent germline transgenerations, principal component analysis (PCA) was used to identify variance and clusters of similarity between the exposed, recovery and control populations. The PCA plots visually indicate the first two principal components (PC1-2) for the F0 generations exposed to the TiO_2_ and Ag NM, with PC1 explaining 96.8% (TiO_2_) and 43.7% (Ag) of the total variance, showing clear separations between each of the differently coated TiO_2_ NM exposures and controls (Figure 4A,B). Figure 4B shows clear separation along the PC1 axis between the control, Ag_2_S, uncoated Ag NMs and Bulk Ag, which was mainly determined by DNA polymerase, NADH and GST, genes associated with DNA repair, oxidative stress, xenobiotic detoxification and energy, respectively [47,48]. PC2 accounted for 23.7% of the variance of the Ag NMs separated by β-actin, CAT and MET, all of which are genes associated with cell maintenance, oxidative stress and metal detoxification [47,48]. Figure 4C shows the three different broods of the F1 intergenerations exposed to the TiO_2_ NM (continuously exposed and recovery). There were clear separations between the intergenerational broods under continuous exposure, with the recovery sets of each differently coated TiO_2_ NMs clustering together towards PC1 (63.3% total variance on PC1, which was split mainly between DNA polymerase, NADH and GST). Interestingly, the recovery generations overlapped much more closely with the control populations relative to the continuously exposed broods, indicating very different responses in these paired exposures.

Figure 4D shows the three different broods of the F1 generations for the Ag NM exposures. The continuously exposed and recovery F1 broods exposed to Ag_2_S clustered together, as did the recovery sets of the Bulk Ag, uncoated and PVP-coated Ag NM exposures (with the exception of the F1B3 PVP Ag recovery set). Variations between the PVP-coated Ag NMs F1B1 and F1B3 sets was observed, while the F1B5 sets show less variation, clustering close to the recovery and Ag_2_S sets. These results indicate that the exposed daphnids separate by their differential gene expression loading profiles (indicated by the arrows on the figures) and individual broods. Figures S10 and S11 show the corresponding F2–4 generations and the interclonal broods (first, third and fifth broods) in the germlines of the F1B1, F1B3 and F1B5 generations. The separation patterns between NM exposure, recovery and brood groupings in the PCA biplots continued in the subsequent generations. The results also show that there is genetic variability between the tolerance of the three F1 broods, which appeared to increase with prolonged maternal exposure for the uncoated and PVP coated Ag NM exposures [28]. The PCA loadings for all germlines and generations following each of the three F1 broods are provided in the Supporting Information, Tables S11–S32.

**Figure 5:**
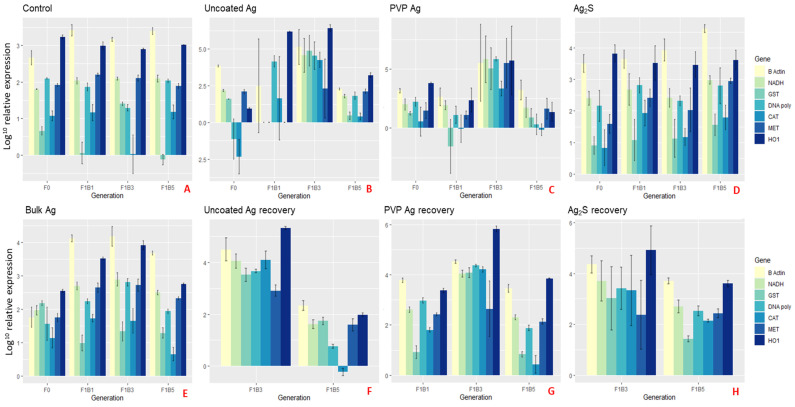
Gene expression barplots of the average relative expression (Log_10_) for parent (F0) *Daphnia* controls (**A**) or exposed to the different Ag NMs (**B–D, F–G**) or bulk silver (**E**), and the relative gene expression levels of the first (F1B1), third (F1B3) and fifth (F1B5) broods born of the F0 Parent in either continuous exposure (**B**–**E**) or removed for recovery (**F**–**H**), determined after 24 h. Error bars show the standard deviation and the NM gene interaction statistics are presented in Table S34 in the supplementary information. *p* ≤ 0.05 was considered statistically significant. Key = Glutathione S-transferase (GST), dehydrogenase (NADH), β-Actin (B-Actin), catalase (CAT), metallothionein (MET), DNA Polymerase (DNA-poly) and heme-oxygenase-1 (HO1). All genes are normalised to 18S ribosomal RNA (18S).

**Figure 6:**
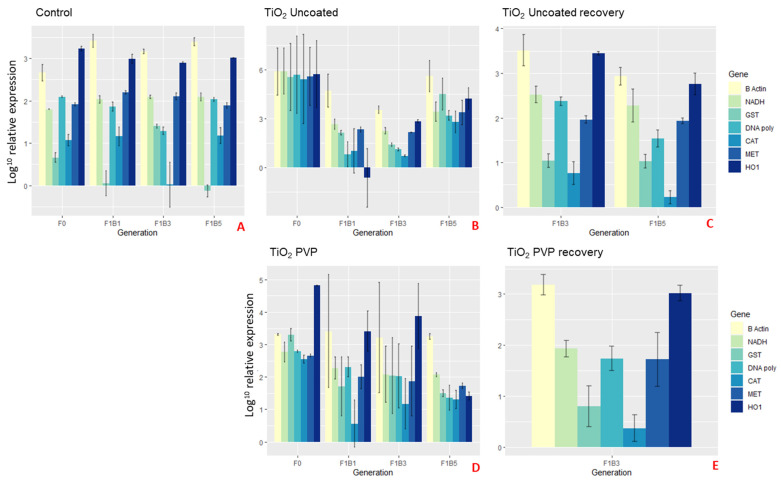
Gene expression barplots of the average relative expression (Log_10_) for parent (F0) *Daphnia* that are (**A**) control, or exposed to the different TiO_2_ NMs (**B–D**), and the relative gene expression levels of the first (F1B1), third (F1B3) and fifth (F1B5) broods born of the F0 parent in either continuous (**B** and **C**) or removed (**C** and **E**) exposure determined after 24 h. Error bars show the standard deviation and the NM gene interaction statistics are presented in Supplemental tables. *p* ≤ 0.05 was considered statistically significant. Key = Glutathione S-transferase (GST), dehydrogenase (NADH), β-Actin (B-Actin), catalase (CAT), metallothionein (MET), DNA Polymerase (DNA-poly) and heme-oxygenase-1 (HO1). All genes are normalised to 18S ribosomal RNA (18S).

Figure 5 and Figure 6 show the average relative gene expression profiles (barplots) of the selected genes compared between the NM exposures for each of the F0 generations and the F1B1, F1B3 and F1B5 broods. Figures S12–S15 show the gene expression profiles (barplots) for the corresponding F2–4 transgenerations of both the continuously exposed and recovery daphnids of the subsequent germlines of the three F1 broods, which further demonstrates that the separation between the different intergenerations (broods 1, 3 and 5) as a result of the increasing maternal adaption to NM-exposure, is maintained in each subsequent generation. 

F0 daphnids exposed to the uncoated Ag NMs had significantly (Figure 5B and Figure S34; *p < 0.05*) increased expression of β-actin (1.4 fold), MET (1.08 fold) and CAT (1.2 fold), and significant down-regulation of GST (0.5 fold), DNA polymerase (2.4 fold) and HO1 (2.1 fold) when compared to the control (Figure 5A). In the F1 generations (F1B1_exp_, F1B3_exp_ and F1B5_exp_) continuously exposed to the uncoated Ag NMs, common upregulated genes across the broods were DNA polymerase, CAT, MET and HO1. These genes were highly expressed in the F1B3_exp_ generation (fold changes of CAT 2.1, MET 1.6 and HO1 2.5 fold) but significantly reduced in the F1B5_exp_ generations (compared to lower fold changes of CAT 0.85, MET -1.1 and HO1 -0.3), showing some adaptation to the NM induced oxidative stress and the energy required for NM detoxification after prolonged maternal exposure (note Table S2 in the Supporting information provides summary details of the main functions of each of the genes analysed). The F1B5_exp_ generation appeared to have the most sensitivity in their physiological (phenotypic) responses with only 20% survival at day 25 and 48% of the daphnids being male. It has been suggested that survivorship is characterised by high mortality early in life, showing the possibility of natural selection pressure on the daphnids phenotype [16]. Therefore, only the most adapted daphnids exposed to the uncoated Ag NMs survived in the F1B5_exp_ generation, as shown by the reduced gene expression levels of key genes involved in oxidative stress, metal detoxification, DNA repair, protein production and cellular maintenance compared to the non-exposed control daphnids. This was also evidenced by the reduced expression in the F1B5_exp_ genes (β-actin -0.6, NADH -1, GST -0.8, and HO1 -0.3) compared with those born earlier in the F1B1_exp_ generation which had a greater fold change compared to the controls (β-actin 1.9, NADH 2, GST 2.2, and HO1 4.2). The F2 expression profiles for the F1B1 germ line also had subtly reduced expression in the F2B5 daphnids compared to the F2B1 daphnids, which was also observed in the F3 and F4 generations. Furthermore, the F1B5 germline also had the same pattern of reduced expression in the latter broods of the F2-F4 generations (Figure S12).

Elevated gene expression was also observed in the F1B3_exp_ of the continuously exposed PVP Ag NMs (Figure 5C), whereas the F1B5_exp_ was significantly reduced in comparison to the daphnids exposed to the uncoated Ag NMs (by ≥ 2-fold in some cases). Daphnids also had increased survivorship, growth and reproductive outcomes in the F1B5 germ line generations compared to the F1B3 germline, further evidencing clonal selection of more tolerant and adaptive genotypes [16].

Increased maternal stress was evidenced by the differences in the gene expression of the three F1 broods in continuous exposure to Ag_2_S NMs (Figure 5D). Unlike the daphnids exposed to the uncoated and PVP coated Ag NMs, the expression of β-actin, NADH, CAT and MET in the latter broods increased gradually compared to the previous brood, as shown by the lower fold changes of F1B1_exp_ for β-actin (1), NADH (1.1), CAT (1.3) and MET (1.1) compared to the higher fold changes of F1B5_exp_ for β-actin (1.3), NADH (1.2), CAT (1.4) and MET (1.5) relative to the controls. In addition to the altered life history traits (survival, reproduction and growth), chronic maternal exposure has increasingly negative effects on key genes upon exposure to the NMs. Previous research suggests that Ag NM toxicity is mediated by oxidative stress leading to mitochondrial damage, lipid damage and cellular apoptosis [48,52,53], and similar effects are evident here with increased expression of all seven genes relative to the controls for the Ag_2_S NMs. The extent of toxicity has been shown to be tuned by the NM surface coating [53,54] shown by the differential responses to the uncoated and PVP coated NMs versus the Ag_2_S NMs in the present study. Ag_2_S NMs were selected to represent environmentally relevant NMs, with lower dissolution potential and thus potentially lower toxicity [55,56,57]. In terms of their impacts on daphnids, we see less damaging effects to the life history traits of growth, longevity and reproduction throughout the exposed generations and germ lines, but strong effects of the Ag_2_S NMs on gene expression when compared to the healthy controls. 

Interestingly, the mothers (F0) exposed to the uncoated TiO_2_ NMs had more than double (β-actin and HO1), triple (NADH, DNA polymerase and MET) or 5-6-fold (CAT and GST) over expressed genes when compared to the controls at the 24 h exposure time point (Figure 6A,B). These results correlated with the overall reduced survival and fitness, whereby energy had been maximised for general homeostasis, oxidative stress and DNA repair in response to the uncoated TiO_2_ NMs exposure [58,59,60]. Although there were no surviving F1 generations post 24 h for each of the three broods born into exposure to the uncoated TiO_2_ NMs (except F1B5 recovery in Figure 6C), we were still able to harvest the neonates prior to death to observe the gene expression levels for each brood. The expression levels for the F1B1 and F1B3 broods were significantly lower than both the control and F0 parent gene expression, however these populations did not survive. This may be due to the maternal exposure time to the NMs, since the first broods (F1B1) and third broods (F1B3) were born after 17 and 30 days exposure, respectively. Adaptations where seen in the F1B5 (and following germ line) once they were removed from the exposure. 

Overall, the results show epigenetic changes and how ‘generational memory’ influences the inter- and transgenerational population dynamics in *Daphnia* ecology. These results show how *Daphnia* invest energy and resources to deal with excessive NM induced stress (reduced survival/growth), which was also shown as phenotypic changes (changes in the gene expression of the observed genes) between the interclonal broods born of the same exposed mother. 

## 3. Materials and Methods

### 3.1. Nanomaterials and Characterization 

Differently surface coated Ag and TiO_2_ NMs were specifically selected, since both materials are routinely used in health and fitness products, and have the most potential for widespread environmental release [61,62]. NMs used in this study include uncoated and PVP-coated TiO_2_ NMs (both supplied by Promethean Particles Ltd, Nottingham, UK), Ag_2_S PVP coated (AppNano Ltd., Barcelona, Spain), uncoated (bare) Ag NMs (Promethean Particles Ltd.) and PVP coated Ag NMs (Amepox Ltd., Łódź, Poland). A bulk Ag particle was also included (Sigma, Dorset, UK).

Using a Malvern Nanosizer 5000 instrument, dynamic light scattering (DLS) was used to measure the hydrodynamic size of the NMs. Transmission electron microscopy (TEM) was used to visualise the NMs, and analysis was performed using a JEOL 1200EX 80kv and a JEOL 1400Ex 80kv Max system. NM solutions were prepared for TEM by depositing 5–10 μL of the NM suspension onto a 300 mesh carbon-coated copper TEM grid (Agar Scientific, Stansted, UK) and allowing it to dry. Primary particle sizes were determined by counting at least 100 NMs. 

### 3.2. Maintenance and Culturing of Daphnia Magna

Initial cultures of *Daphnia magna Straus* [63] were maintained using pools of 3rd broods of Bham2 strain (Clone Type 5) and were sustained in a temperature controlled environment (20°C) with 12 h light and dark cycles. *Daphnia* were cultured in HH combo medium [64] which is designed to match the total hardness of water found in the environment without any natural organic matter. Details of the medium composition are given in the Supplemental Information (Table S1/S1A/S1B). The medium was refreshed twice weekly to ensure healthy culture maintenance. Cultures (and study daphnids) were fed *Chlorella vulgaris* algae daily (0.5 mg carbon for days 0–5 and 0.75 mg carbon from day 5). 

### 3.3. Immobilization Tests 

OECD 202 immobilization tests [65] were conducted to derive the 48-h effect concentration (EC) values. For the Ag NMs (EC_30_) values of 20 µg L^−1^ for uncoated and PVP-coated Ag NMs, and 100 µg L^−1^ for Ag_2_S, and (EC_5_) values of 5 mg L^−1^ for uncoated and PVP-coated TiO_2_ NMs (Figure S1) were established. There is a difference between using environmentally relevant concentrations and the ECs; the justification for using ECs is that environmental risk assessments of chemical exposure hazards are assessed by characterizing the effects in biological receptors, and thus some level of response was required. The acute EC_30_ values of the TiO_2_ NMs had been found previously to result in F0 death during the chronic exposures and thus the EC_5_ was used instead [66].

### 3.4. Technical Design

**Figure 7:**
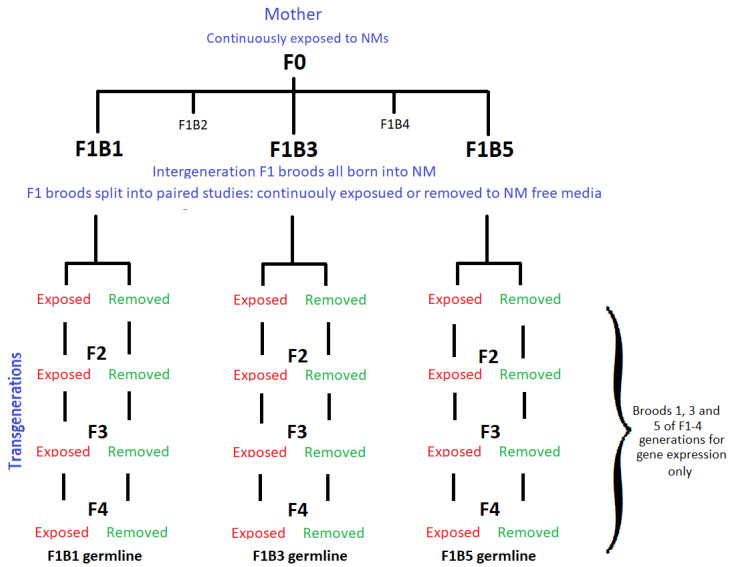
Multigenerational design showing the exposed and recovery generations after the F0 parental exposure. The intergeneration’s of the F1 broods born of F0 (F1B1, F1B3 and F1B5) are then monitored over the transgenerations, following each of the germlines from F2-F4 thereafter for each brood. Note, the F1 recovery generations are born into NM-exposure but removed within 24 h of birth, to assess the recovery potential and whether there are epigenetic effects from the maternal exposure. The red text shows the exposed transgenerations within each germline during continuous exposure. The green text shows the exposed transgenerations within each germline in the recovery generations.

To assess the maternal impact of continuous NM exposure on the interclonal broods, the first (F1B1), third (F1B3) and fifth broods (F1B5) born of the F0 parent, were split at birth into paired studies to monitor their following germlines (Figure 7). These paired studies split the neonates from each brood into either a continuous exposure (at the same NM concentrations as the mother (F0)) or daphnids removed to NM-free medium, i.e., to recover. The three intergenerational F1 broods (broods 1, 3 and 5) were further monitored for transgenerational inheritance within their germlines for expression of key stress response genes, which were compared to the intergeneration differences. The F1B3 transgenerational response has also been analysed in more detail [24,66,67] in order to identify the modulating toxic effects of chronic exposure to both pristine and long term environmentally aged NMs, resulting in a range of toxicological outcomes including ageing phenotypes. 

### 3.5. Survival, Growth and Reproduction

Each of the F1 broods (F1B1, F1B3 and F1B5) and their subsequent F2, F3 and F4 transgenerations were monitored daily for survival, egg production and neonate release. Measurements of body length (growth) were taken every 3 days (between days 3–24) in accordance with moulting of the carapace [36]. Total body length was determined measuring from the apex of the helmet to the base of the apical spine using a Nikon (Japan) stereomicroscope, model SMZ800 Digital Sight fitted with a D5-Fi2 camera using NIS Elements software. 

### 3.6. Gene Expression

To compare a fixed time point across all generations and the different exposures, pools of 20 neonate daphnids were sampled after 24 h exposure to the NMs in the F0 generation, and neonates in the following germlines and generations were also monitored at the same 24 h (exposure to or removal from NMs) time point. Daphnids were euthanized using liquid nitrogen and were mechanically homogenized using a Precellys 24 instrument (Bertin Technologies) (with approximately 30 beads each tube), using 2 cycles of a 30 s pulse at 6000 pulse speed. Samples were then stored at −80°C until extraction.

An Agencourt RNAdvance Tissue Kit (Beckman Coulter A47943) using paramagnetic bead-based technology was used for total RNA isolation and purification. RNA isolation was performed using a Beckman Coulter Biomek FxP. A total of eight genes were selected for target-specific amplification using a mix of previously published primer sequences (Table S2), including; Glutathione S-transferase (GST), dehydrogenase (NADH), β-actin (B-Actin), catalase (CAT), metallothionein (MET), DNA Polymerase (DNA-poly), heme-oxygenase-1 (HO1), and 18S ribosomal RNA (18S). Primer sequences were also checked using NCBI primer blast software (https://www.ncbi.nlm.nih.gov/gene) for the probability of amplifying nonspecific products. A Onestep qPCR kit (Qiagen) was used in accordance with the manufacture’s guidance for reverse transcription (Tables S3–S5). Gene expression was conducted using Flex Six Integrated Fluidic Circuit (IFC) Delta Gene Assay (72 × 72) in combination with a HX Prime (153x) system and a Fluidigm BioMark (Standard) Real time PCR instrument, as per the manufacture’s recommended protocol (Tables S6 and S7). An in-depth description of the methodology can be found in Section S1.3 of the Supporting Information. All data are deposited in the Gene Expression Omnibus (GEO). 

### 3.7. Statistical Analysis

All experiments were performed in triplicate, and the data was recorded as the mean with standard deviation. In all analyses, a *p*-value < 0.05 was considered statistically significant. Gene expression levels were normalized to 18S expression [68]. Statistical significance of changes in gene expression (Table S34) and Principal Component Analysis (PCA) plots were computed in RStudio to identify the significance of parameters and reported variance (Tables S11–S32). Analysis of growth was assessed using Student’s *t*-test to detect any significant difference between the control, treated and recovery groups (Table S34).

## 4. Conclusions

The maternal exposures provide strong evidence that the tested Ag and TiO_2_ NMs are toxic to *Daphnia*, which showed reduced fitness, survival and reproduction. In addition, maternal exposure to the tested NMs further impacted the offspring performance across all three F1 intergenerational broods, which showed altered growth rates, survivorships and inhibited reproduction in both continuously exposed and recovery groups. Clear heredity phenotypical responses from the increased maternal stress over time were highlighted, with the most sensitive broods being the F1B1 and F1B3 germlines for the PVP and uncoated Ag NMs exposures. Adaptive and compensative mechanisms, driven by the prolonged maternal exposure, were observed in the F1B5 broods, with increased survival in the exposed populations for uncoated and PVP-coated Ag NMs, which was further evidenced in the F2 and F3 transgenerations of this germline. Central to these observations is the determination of the heritability tolerance, whereby chronic exposure of the F0 mothers’ results in natural selection of the strongest surviving daphnids in the F1 broods. However, prolonged maternal exposure to Ag_2_S and PVP-coated TiO_2_ NMs caused increased sensitivity of the F1B5 germlines with reduced overall survival. 

Gene expression analysis gave insights into the observed phenotypical difference between each of the broods, and particle specific impacts as a direct response to prolonged maternal exposure. In addition, the gene expression responses may provide an indication of how other organisms are similar or differ in their response to NM exposures. 

Our results show how daphnids respond to NM-induced stress, and how the maternal effects show trade-offs between growth, reproduction and survivorship. Moreover, the sensitivity is maintained in the germlines of the broods as evidenced by the weaker F1B1 broods and more adapted F1B5 broods (in most cases) which correlated with increased duration of maternal exposure to the NMs. 

Transgenerational responses of multiple germlines had a direct link to maternal exposure duration to what are considered “sub-lethal” effect concentrations of the tested NMs (based on acute tests), which chronically present as lethal to both the F0 daphnids and in many cases to the offspring. It is notable that although multi-generational effects are not yet routinely studied as part of standardised testing for regulatory assessment, although the evidence presented in this study suggest that they should be. This information may help to fine-tune environmental risk assessments and assessment of NMs impacts on environmental ecology and provide further evidence to support a revision of the chronic daphnia reproductive tests to include effects on the F1 (and potentially F2) generations under both continuously exposed and recovery conditions. 

## Figures and Tables

**Table 1:** Summary characterization data for TiO_2_ and Ag NMs. From Ellis et al., 2020 [24].

Identifier	Pristine TEM Individual Particle Size (nm)	Pristine DLS Particle Size (nm)	Polydispersity Index (PDI)	Surface Coating
**TiO_2_ Uncoated**	9 ± 2	207 ± 11	0.5	Bare
**TiO_2_ PVP**	9 ± 2	311 ± 43	0.4	PVP_10_
**Ag_2_S**	44 ± 14	299 ± 6	0.4	PVP_10_
**Ag PVP**	18 ± 11	260 ± 180	0.3	PVP_10_
**Ag uncoated**	61 ± 36	120 ± 30.5	0.5	Bare

Note: The TEM sizes are reported as the individual nanomaterial (NM) sizes (primary particle size). PDI = polydispersity index.

## Supplementary Materials

Supplementary Materials can be found at https://www.mdpi.com/1422-0067/22/1/15/s1. These include the detailed description of the experimental steps including: details of the media (Table S1), the range finding study (Figure S1), the gene expression (Tables S2–S7), and additional results including nanomaterials characterization (Figure S2), multigenerational longevity data for each of the three germlines (Figures S3–S5), growth (Figure S6), reproduction shown as total neonates for the three germlines (Figures S7–S9 and Tables S8–S10), and gene expression (Figures S10—S15). Tables S11–S32 show the PCA loadings, and Table S33 gives the output from the 2-tailed *t*-test on daphnid growth compared to controls for Ag NPs. Table S34 contains the statistical data for the gene expression analysis.

## Abbreviations

Ag	Silver
Ag_2_S	Silver Sulphide
B-Actin	β-Actin
CAT	catalase
DLS	dynamic light scattering
DNA-poly	DNA Polymerase
F1B1	Germline born of the F1 generation first brood
F1B3	Germline born of the F1 generation third brood
F1B5	Germline born of the F1 generation fifth brood
GEO	Gene Expression Omnibus
GST	Glutathione S-transferase
HO1	heme-oxygenase-1
MET	metallothionein
NADH	Dehydrogenase
NMs	Nanomaterials
NOM	Natural organic matter
OECD	Organisation for Economic Co-operation and Development
PCA	Principal Component Analysis
qPCR	quantitative Polymerase Chain Reaction
RNA	Ribonucleic acid
SPFs	Sun protection factor
TEM	Transmission electron microscopy
TiO_2_	Titanium dioxide
18S	18S ribosomal RNA
